# *Argonaute1* and *Gawky* Are Required for the Development and Reproduction of Melon fly, *Zeugodacus cucurbitae*

**DOI:** 10.3389/fgene.2022.880000

**Published:** 2022-06-23

**Authors:** Momana Jamil, Shakil Ahmad, Yingqiao Ran, Siya Ma, Fengqin Cao, Xianwu Lin, Rihui Yan

**Affiliations:** ^1^ School of Life Sciences, Hainan University, Haikou, China; ^2^ School of Plant Protection, Hainan University, Haikou, China; ^3^ Key Laboratory of Green Prevention and Control of Tropical Plant Diseases and Pests, Ministry of Education, School of Plant Protection, Hainan University, Haikou, China; ^4^ Hainan Yazhou Bay Seed Lab, Sanya, China

**Keywords:** *Zeugodacus cucurbitae*, Argonaute, functional analysis, RNA interference (RNAi), ovarian development, mortality

## Abstract

Argonaute family genes encode a highly conserved group of proteins that have been associated with RNA silencing in both animals and plants. This study investigates the importance of microRNA biogenesis key regulators *Argonaute1* (*Ago1*) and *Gawky* genes in the post-embryonic and ovarian development of the melon fly, *Zeugodacus cucurbitae*. The expression levels of these genes were mapped in all developmental stages and different adult tissues. Their roles in development were investigated using RNA interference (RNAi) *via* two different dsRNA delivery techniques. Embryo microinjection and oral feeding of third instar larvae successfully knocked down and greatly reduced the expression level of the target genes. Additionally, ex vivo essays revealed the stability of dsRNA in food was sufficient for gene silencing, although its integrity was affected in midgut. A wide range of phenotypes were observed on pupation, segmentation, pigmentation, and ovarian development. RNAi-mediated silencing of *Gawky* caused high mortality and loss of body segmentation, while *Ago1* knockdown affected ovarian development and pigmentation. Developmental abnormalities and ovarian malformations caused by silencing these genes suggest that these genes are crucial for viability and reproductive capacity of *Z. cucurbitae*, and may be used as potential target genes in pest management.

## Introduction

Argonaute (Ago) is a unique class of proteins that are required for small non-coding RNA (sncRNA)-mediated gene regulation. sncRNAs such as small interfering RNA (siRNA), micro-RNA (miRNA), and Piwi-interacting RNA (piRNA) have rapidly emerged as key regulators of gene expression in the last decade (Aalto and Pasquinelli 2012). Numbers of the Ago family members differ in insect species, for example, four are present in *Bombyx mori* and five in *Tribolium castaneum* and *Drosophila melanogaster* (Ago1, Ago2, Ago3, Aubergine, and Piwi) (Okamura et al., 2004; Zhu et al., 2013). Ago proteins Ago1 and Ago2 bind with miRNA and siRNA (and other associated factors) to form the RNA-induced silencing complex (RISC) which induces degradation or translational inhibition of complementary messenger RNA (mRNA), while Ago3, Aubergine, and Piwi cooperate to process piRNAs (Hutvagner and Simard 2008). piRNAs play a critical role in maintaining the repression of transposable elements (TEs) and germline integrity across generations (Blumenstiel et al., 2016). Furthermore, target mRNA degradation through miRNA is affected by cytoplasmic foci named P-bodies (processing bodies) (Sen and Balu 2005). These P-bodies accumulate the protein factors (made up of exonucleases and decapping enzyme complex XRN1 and DCP1: DCP2, respectively) required for mRNA degradation from 5′-3′ direction (Behm et al., 2006; Niaz et al., 2018). P-bodies integrity is maintained by GW182 protein (Gawky) which is rich in glycine (G) and tryptophan (W) residues in its N terminal region and required for the interaction with Ago1 (Ingelfinger et al., 2002; Behm et al., 2006; Eulalio et al., 2007). There are three paralogs of Gawky (TNRC6A, TNRC6B, and TNRC6C) in vertebrates and one in insects (Schneider et al., 2006). Two orthologs of Gawky, AIN1 and AIN2, are required to work together with Ago proteins (ALG-1 and ALG-2) and miRNA in *Caenorhabditis elegans* (Ding and Grosshans 2009; Haskell and Zinovyeva 2021). Mutations in these orthologs suppress the silencing efficiency and cause developmental timing problems in *C. elegans* (Ding et al., 2005). In *D. melanogaster*, Ago1 and Gawky are involved in miRNA-guided mRNA silencing (Hanyu et al., 2019; O'Brien et al., 2018), and impaired miRNA function was observed in Gawky or decapping enzyme complex-depleted S2 cells (Rehwinkel et al., 2006). GW182 depletion impairs miRNA function and mRNA degradation in human cells (Jakymiw et al., 2005; Meister et al., 2005).

The melon fly, *Zeugodacus cucurbitae*, is one of the most destructive pests of cucurbit crops, causing severe damages to the fruit through oviposition, punctures, and larval development (Bhupen and Ispita 2020). Physical (traps, barriers, *etc*.) and chemical treatments are currently used to control the pest (Stejskal et al., 2021). However, the frequent use of chemical pesticides has resulted in resistance development and negative consequences for the environment and human health (Ansari et al., 2014; Kumar and Singh 2015; Sharma et al., 2020). Therefore, focusing on the functions of key genes involved in development and reproduction is necessary for future pest control. In previous research, the knockdown of imaginal disc growth factor (*IDGF*) genes caused developmental defects in *Z*. *cucurbitae* (Ahmad et al., 2021). However, to control this destructive pest, the identification of lethal genes is encouraging. We have been intrigued by insect *Ago1* and *Gawky* genes due to their roles in the developmental process, such as ovarian development and viability (Brennecke et al., 2003; Song et al., 2013). Previous studies have shown that Ago1 and Ago1-dependent miRNAs are crucial for reproduction in many insects. Ago1 and its miRNA biogenesis partners are indispensable for oocyte formation, self-renewal of germline stem cells (GSCs), and female germline cell division (Park et al., 2007; Azzam et al., 2012). A previous work has demonstrated that *Ago1* deletion mutants of *D. melanogaster* was unable for self-removal of GSCs in ovaries. *Ago1* mutations cause mitotic abnormalities during early embryogenesis of *Drosophila*, indicating that it plays a vital role in development (You et al., 2019b; Heyt and Thakur 2021). The *Ago1* studies in *Locusta migratoria* and *Bactrocera dorsalis* have shown that loss of *Ago1* affects ovarian growth by decreasing vitellogenin expression (Yang et al., 2021). Yet there is no study about the role of *Ago1* in the ovary development of *Z. cucurbitae*. The regulation of ovarian development is an essential physiological process which is critical to reproduction and population growth. Low fertility and fewer offspring lead to easier insect control because of a decreased population level.

RNA interference (RNAi) has been widely applied as a loss-of-function approach for exploring gene function. However, RNAi efficiency varies in different tissues or at different developmental stages of every species (Terenius et al., 2011; Mamta et al., 2017; Silver et al., 2021). Several RNAi genes govern the RNAi process and regulate RNAi efficiency. Identifying and characterizing RNAi genes might help us better understand their functions in development and implement RNAi-based pest control techniques. This study investigates the physiological role of *Ago1* and *Gawky* genes that may be used as new pesticide targets. In this study, we have 1) investigated the expression patterns of target genes at various developmental stages and tissues; 2) performed functional verification of *Ago1* and *Gawky* in reproduction; 3) performed gene silencing *via* two techniques, injection and oral feeding; and 4) evaluated dsRNA stability in the midgut and food for possible degradation upon oral delivery. Our results indicate that *Ago1* and *Gawky* genes are essential for reproduction and viability, and may contribute significantly to pest control.

## Materials and Methods

### Insect Rearing

*Z*. *cucurbitae* were initially collected from Haikou, Hainan Province, China, and reared in the laboratory for many generations. The insect larvae and adult were fed on an artificial diet (Liu et al., 2020), and a 1:3 yeast powder and sugar diet, respectively, and maintained under 26°C, 14 h light: 10 h dark photoperiod at 70% relative humidity. Grown larvae were shifted to wet sand prior to pupation, and then pupae were transferred to cages until adult emergence.

### Phylogenetic Tree Construction and Domain Architecture

Nucleotide sequences of *Ago1* (Gene ID: 105216341) and *Gawky* (Gene ID: 105220406) were obtained from the *Z. cucurbitae* genome (Sim and Geib 2017) by BLAST in NCBI using *Ago1* and *Gawky* genes of *D. melanogaster* as queries. Both genes were then verified by reverse transcription PCR (RT-PCR) and sequencing. The SMART (http://SMART.embl-heidelberg.de) and ExPASy Prosite SCAN (https://prosite.expasy.org/scanprosite/) were used to analyze the conserved domains and functional sites. The phylogenetic tree was constructed through the neighbor-joining method by taking Tephritidae and Drosophilidae as model families with 1,000 bootstrap repetitions in MEGA-X.

### Collection of Samples From Different Developmental Stages and Tissues

To examine the expression profiles of *Ago1* and *Gawky*, samples were collected from different developmental stages: 1^st^ instar larvae (1L), 2^nd^ instar larvae (2L), 3^rd^ instar larvae (3L), pupae and adults, and various tissues: head, ovary, testis, fat body, and midgut from adults at 1, 2, 5, and 7 days old. Flies were dissected separately in 1 × PBS (pH 7.4) under a binocular stereoscope (Olympus SZX12, Tokyo, Japan). For each biological replicate, ovaries from females at 1–2 days old contained 20 pooled flies, whereas ovaries from females at 5 and 7 days old had 10 pooled flies. All samples included three independent biological replicates.

### cDNA Synthesis and dsRNA Preparation

Total RNA was isolated from 3^rd^ instar larvae using a TRI Reagent^®^ (Sigma, United States). cDNA was reversely transcribed from 1 μg of the total RNA template using the SuperScript III First-Strand Synthesis kit (Takara, Dalian, China) and used to amplify the open reading frame (ORF) of target genes. ORF was amplified using PrimeSTAR® HS DNA Polymerase (Takara, Dalian, China) and cloned into the pMD™18-T vector (Takara, Dalian, China) for sequencing. For dsRNA preparation, T7 promoter sequences were introduced to the 5′ ends of the forward and reverse primers of *Ago1* and *Gawky* genes (Supplementary Table S4). A green fluorescent protein (GFP) fragment was amplified from the pCAMBIA1303 expression vector. PCR program was performed as follows: 95°C for 3 min (m), 32 cycles of denaturing at 95°C for 30 sec (s), annealing at 56–58°C for 30 s, and extension at 72°C for 28 s with a final incubation at 72°C for 10 m. PCR products were electrophoresed on a 1% agarose gel, extracted, purified, and cloned into pMD™18-T vector. Positive clones were confirmed by PCR using M13 forward and gene-specific reverse primers. Plasmid DNA was extracted from positive clones using the Sangon Plasmid miniprep kit (Sangon Biotech, Shanghai, China) and sequenced at Sangon Biotech (Shanghai, China). dsRNA was synthesized using corresponding plasmids as the template with the MEGAscript RNAi kit (Promega, United States). Nuclease-free water was used for dsRNA elution. dsRNA was quantified on a NanoDrop 2,000 spectrophotometer at 260 nm, and integrity was determined using gel electrophoresis. dsRNA was diluted to 1 μg/μl in nuclease-free water.

### Quantitative Real-Time PCR (qRT-PCR) for Expression Analysis

qRT-PCR was used to evaluate the effects of dsRNA treatments on *Ago1* and *Gawky* expression levels in various tissues and developmental stages of *Z*. *cucurbitae*. qRT-PCR was performed using gene-specific primers (Supplementary Table S4). The amplification efficiency of the primers was first confirmed by a standard curve based on a 4-fold cDNA dilution series. qRT-PCR was performed in a 10 μl reaction (5 μl of 2x SYBR Green qPCR Supermix Plus, 0.5 μl of each pair of primers, 0.5 μl of cDNA, and 3.5 μl of distilled water). The qRT-PCR program was conducted as follows: 95°C for 30 s, followed by 40 cycles of 95°C for 5 s, 60°C for 10 s, and 72°C for 15 s in 96-well plates on the Analytik Jena qPCR system. The elongation factor 1 alpha (*EF1*α) and *Actin* were used as an internal reference to normalize the relative transcript levels of cDNA. The transcript levels were quantified using the 2^–ΔΔCT^ method (Livak and Schmittgen 2001).

### dsRNA Degradation in Midgut Juice and Artificial Diet

To check the dsRNA stability in artificial diet and midgut, an *ex vivo* dsRNA degradation assay was performed. Midguts were extracted from 12 adult insects. Dissection was performed in 1x PBS under a microscope by first immobilizing the insects on ice for 5 m. All the unwanted tissues were removed, and midguts were collected in a pre-chilled Eppendorf tube placed on ice and then centrifuged at 13,000 rpm for 12 m; 2 μl supernatant was carefully transferred and mixed in 25 μl of dsRNA (1 μg/μl); 2 μl ddH_2_O was used in mimic control instead of midgut juice, and both samples were incubated at 26°C. About 5 μl of the sample was taken off from both reaction tubes at 0, 15, 30, and 60 m, and placed at −80°C to stop the enzymatic reaction. dsRNA integrity was evaluated on 1% agarose gel. To check the stability of dsRNA in the artificial diet, 20 μl of dsRNA (1 μg/μl) was mixed with a 2 g artificial diet (Liu, et al., 2020). The nuclease-free water was used as a control in food instead of dsRNA. After 1, 24, and 48 h, dsRNA stability was checked by re-dissolving the food in 10 μl ddH_2_O and running on agarose gel for 25 m.

### Feeding Assay

The dsRNA feeding assay consisted of the treatments (ds*Ago1* and ds*Gawky*) and the control group (dsGFP). Early 3^rd^ instar larvae and the control group were fed with an artificial diet mixed with gene-specific dsRNA and dsGFP, respectively. Three biological replicates were performed for each group. Each replicate contained 60 larvae, 2 g of artificial diet, and 20 μl of 1 μg/μl dsRNA. The food of the control group had the same ingredients, except gene-specific dsRNA. Insects were fed with dsRNA for 24 h, and then shifted to the new diet with the same dsRNA for another 24 h. For each replicate, three larvae of 12, 24, and 36 h post-feeding were used for RNA extraction to analyze the knockdown efficiency. The silencing efficiency of the target genes was tested using qRT-PCR. The experimental setup is described in Supplementary Figure S1A.

### Microinjection Assay

For microinjection, 1 μg/ul dsRNA solution of each gene was injected into embryos (usually 1.5 μl is used to fill the injection needle for 300–400 eggs) using FemtoJet 4i microinjector (Eppendorf, Hamburg, Germany). Three biological replicates were performed for the control and treatment groups, where each replicate contained 300 eggs injected with gene-specific dsRNA or dsGFP. To determine gene silencing efficiency, 25 eggs of 12 and 24 h post-injection and 25 1^st^ instar larvae of 36 h post-injection were used for RNA extraction, and qPCR was performed for gene expression analysis. The experimental setup is described in Supplementary Figure S1B.

### Phenotype Observation and Fecundity Analysis

After RNAi treatment with dsRNA injection and feeding, ovaries and testes of 14-day-old female and male adults from each treatment and control group were dissected in 1 × PBS (pH 7.4). Ovary and testis phenotypes were photographed using a Leica M205A stereomicroscope (Leica Microsystems, Wetzlar, Germany). To explore the RNAi effects of *Ago1* and *Gawky* genes on female and male fertility, the virgin females of 9 days old were individually crossed with two virgin wild-type (WT) males of the same age. Each male was paired with two virgin WT females of the same age in a courtship chamber for 5 days. Flies were provided with yeast and sugar to promote egg-laying, and cucumber slices were provided after 24 h for females to lay eggs on it. Oviposition was examined by counting the number of eggs laid by the female flies. Eggs were transferred to artificial food and incubated at 26°C. To assess the fecundity, the number of hatched larvae was counted and the egg hatchability was calculated. Ten females and males were used from each replicate with three repetitions for ds*Ago1.* For ds*Gawky*, five females and males were used from each replicate with three repetitions*.*

### Statistical Analysis

Statistical analysis of differences in mRNA expression, mortality, and fecundity was performed using the GraphPad Prism software package (GraphPad Software Inc., San Diego, CA, United States). The statistical significance of differences between means of each group was assessed using a one-way analysis of variance followed by Tukey’s honestly significant difference (HSD) test.

## Results

### Phylogenetic Analysis and Domain Architecture

**FIGURE 1:**
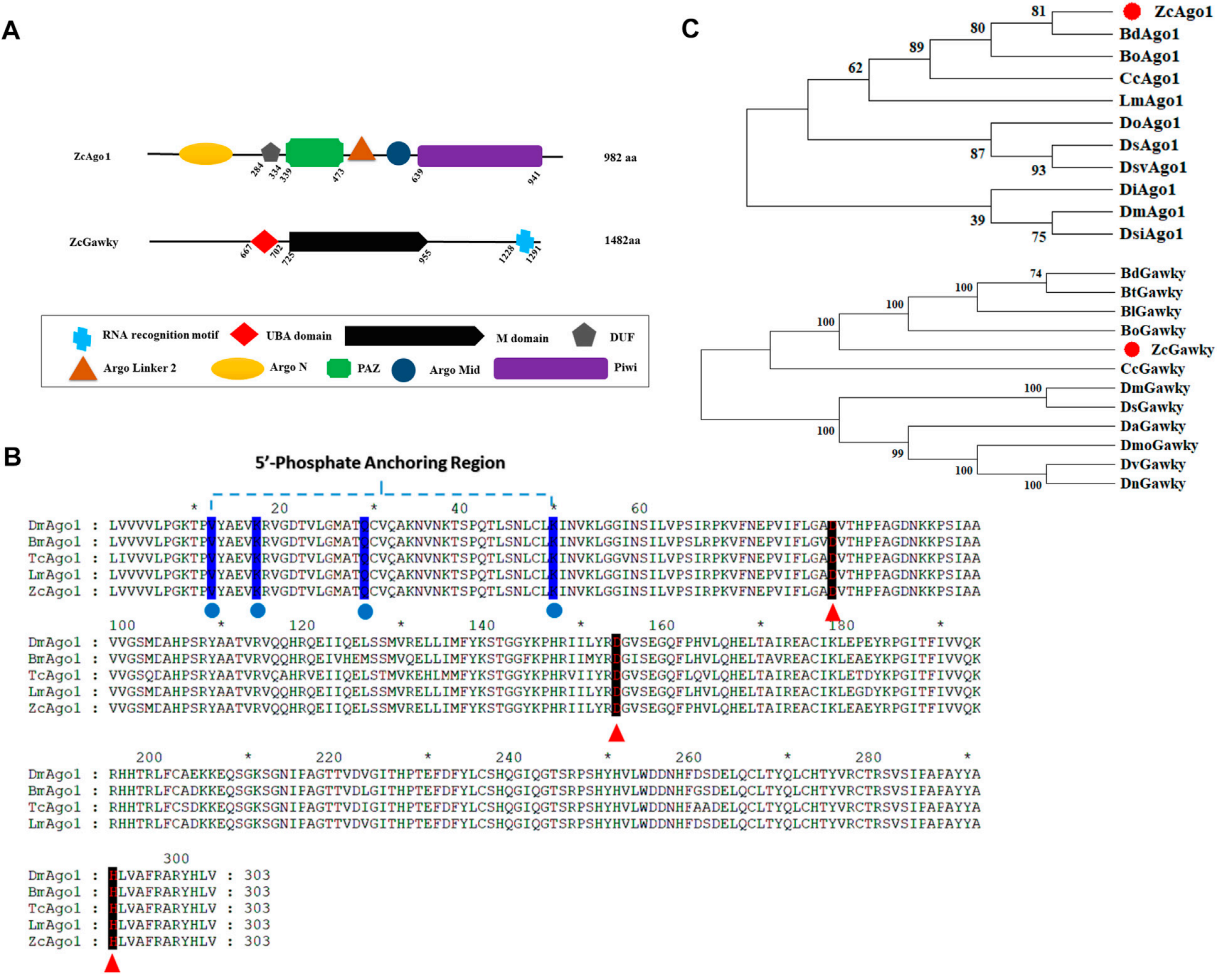
Bioinformatics analysis of Ago1 and Gawky proteins in *Z. cucurbitae*. **(A)** Domain architectures of ZcAgo1 and ZcGawky. Both proteins are involved in miRNA-mediated mRNA silencing. Ago1 contains the PAZ and PIWI domain, while Gawky contains UBA (ubiquitin associated, M (mid) domain, and RRM (RNA recognition motif). **(B)** Motif analysis of Ago1. Comparison of the characteristic PIWI domains in Ago1 proteins from different species. The protein sequences include ZcAgo1 (XP_011189086.1), Ago1 from *D. melanogaster* (DmAgo1: NP_523734), *B. mori* (BmAgo1: BAF73719.1), *T. castaneum* (TcAgo1: CDW23160.1), and *L. migratoria* (LmAgo1: KF006338). The conserved amino acid residues involved in 5′ end recognition sites are marked with blue color and the conserved Asp, Asp, and His (DDH) triad residues are pointed out with red arrowheads. **(C)** Phylogenetic tree for Ago1 and Gawky from different insect species. MEGA-X was used for tree construction following the neighbor-joining method with 1000 bootstrap repetitions per test re-sampling. The level of bootstrap support for each branch is represented with numbers at the node. Gene accession numbers and full names are shown in Supplementary Table S1.

After PCR amplification and sequencing of *Ago1* and *Gawky* genes from 3^rd^ instar larvae of *Z. cucurbitae*, the sequence analysis showed that *Ago1* and *Gawky* contain 982 and 1,482 amino acid residues, respectively. The predicted protein structures using SMART software showed that Ago1 has a DUF domain of an unknown function besides conserved PAZ and PIWI domains (Figure 1A). RNase H-like PIWI domain is consistent with Ago proteins which give them slicer activity. Multiple sequence alignment of the PIWI domains indicated that the 5′ phosphate anchoring region, Aspartate, Aspartate, and Histidine (DDH) motif, is highly conserved in *D. melanogaster*, *B. mori*, *T. castaneum*, and *L. migratoria* (Figure 1B; Supplementary Figure S2), suggesting that Ago1 is a member of the Ago family. *Z. cucurbitae* has just one Gawky protein, similar to *D. melanogaster* (Perconti et al., 2019). Both N and C terminals and mid-region of the *Z. cucurbitae* Gawky protein are also rich in glycine (G) and tryptophan (W). N terminal of the Gawky protein facilitates its interaction with the PIWI domain of Ago1, and RRM (RNA recognition motif) targets mRNA and contributes to its repression (Behm-Ansmant et al., 2006). Phylogenetic analysis of Ago1 and Gawky proteins revealed that *Z*. *cucurbitae* formed a clade with other examined insect species. Ago1 of *Z. cucurbitae* (ZcAgo1) showed the highest similarity with *B. dorsalis* Ago1 protein (BdAgo1), and *Z. cucurbitae* Gawky protein (ZcGawky) also grouped together with its counterparts from *Bactrocera* genus (Figure 1C).

### Temporal and Spatial Expression of *Ago1* and *Gawky* Genes in *Z. cucurbitae*

**FIGURE 2:**
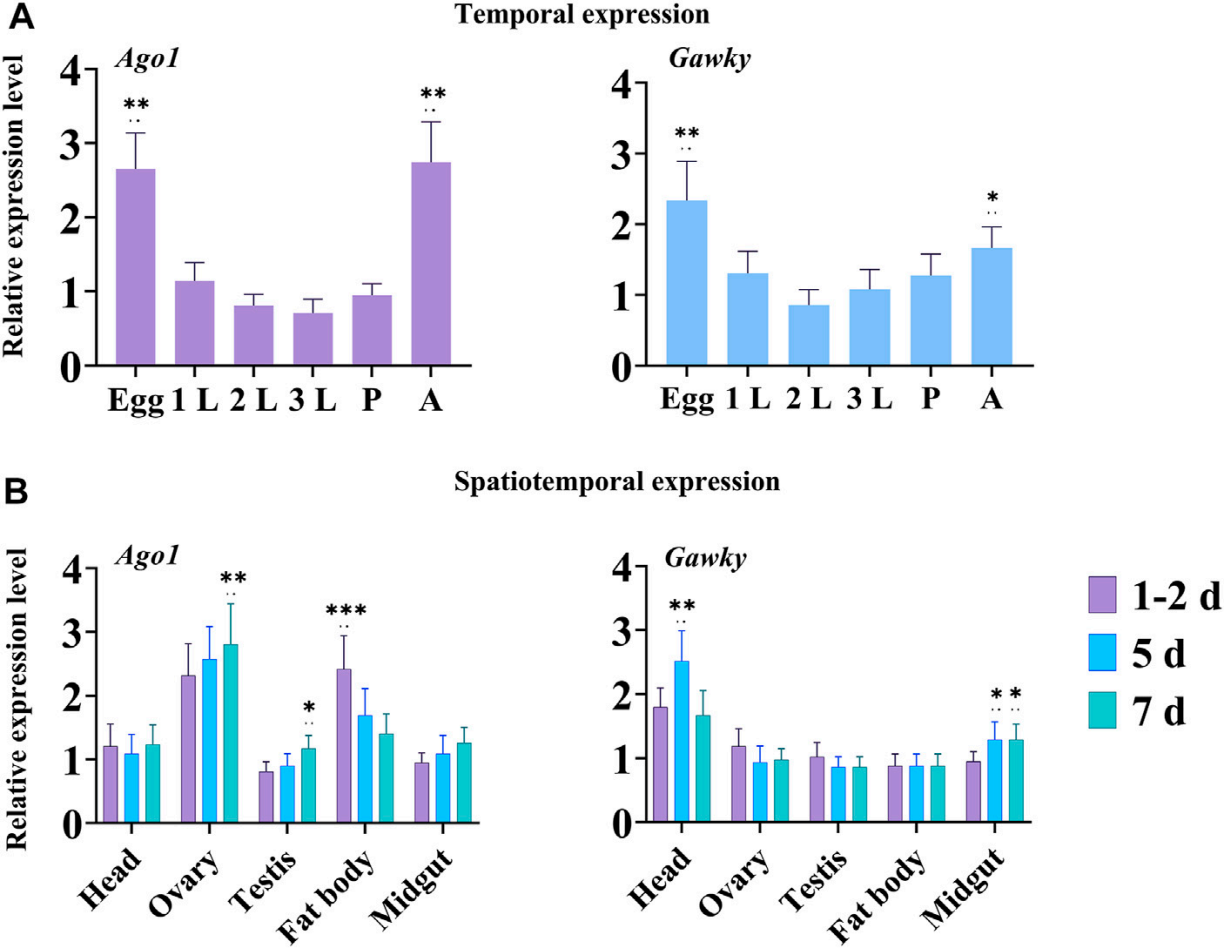
Temporal and spatial expression of *Ago1* and *Gawky* in *Z. cucurbitae.* **(A)** Relative expression levels of *Ago1* and *Gawky* genes in different developmental stages. Egg, 1^st^ instar larvae (1L), 2^nd^ instar larvae (2L), 3^rd^ instar larvae (3L), pupae (P), and adult (A). **(B)** Relative expression levels of *Ago1* and *Gawky* in different tissues. Samples were collected from the head, ovary, testis, fat body, and midgut of ten flies at 1–2 days old, and of eight flies at 5 and 7 days old in each replicate. The bars represent 2^–ΔΔCT^ values (± SD) normalized to the geometrical mean of the expression of two housekeeping genes. The asterisks above the bars designate significant differences (*p < 0.05; **p < 0.01; ***p ≤ 0.001).

The expression levels of the two genes *Ago1* and *Gawky* were measured using qRT-PCR in different developmental stages and five tissues of *Z. cucurbitae* (Figures 2A,B). The temporal expression showed that *Ago1* and *Gawky* were highly expressed in embryos and adults of *Z. cucurbitae* (Figure 2A). In tissue-specific expression analysis, the maximum relative abundance for *Ago1* was recorded in ovaries from females of 5 and 7 days old, followed by fat bodies from adults of 1–2, and 5 days old. Interestingly, the expression of *Ago1* was a little higher in the fat bodies of a 1-2-day-old adult than in the ovaries of females in the same age. For the flies from day 5 to 7, the *Ago1* expression reduced in the fat body but increased in the ovary. *Gawky* showed the highest expression in the head of 5-day-old adult flies, compared to other tissues (Figure 2B). These temporal and spatial expression profiles suggest general roles of *Ago1* and *Gawky* in the whole body of *Z. cucurbitae* and in the female reproductive system.

### dsRNA Stability in Artificial Food and Midgut Juice of *Z. cucurbitae*

To explore the functions of *Ago1* and *Gawky*, we injected and fed dsRNA to embryos and larvae, respectively. Before that, we first checked dsRNA integrity in artificial food to determine how often food needed to be changed with the fresh one to ensure that larvae were exposed to stable dsRNA. 3^rd^ instar larvae were fed with food containing 20 µl of 1 μg/μl dsRNA. Diet samples taken at 1, 24, and 48 h were separated on 1% agarose gel to check the stability. As shown in Supplementary Figure S3A, dsRNA remains stable in the artificial diet for 24 h, and a weak level of dsRNA exists until 48 h. This indicates that enough stable dsRNA was available to larvae throughout the exposure. On the other hand, an *in vitro* study measuring the integrity of dsRNA in midgut juice revealed that dsRNA was stable for the first 15 m, then poorly detectable after 30 m, and finally completely degraded after 60 m at room temperature. These results indicate that no matter dsRNA degraded after 15 m in midgut juice due to the presence of nucleases, stable dsRNA was constantly available in food for more than 24 h to target the relevant genes. In contrast, dsRNA remains stable in the control (Supplementary Figure S3B). Therefore, we fed larvae with a new diet containing dsRNA every 24 h.

### RNAi-Mediated Knockdown of Target Genes

**FIGURE 3:**
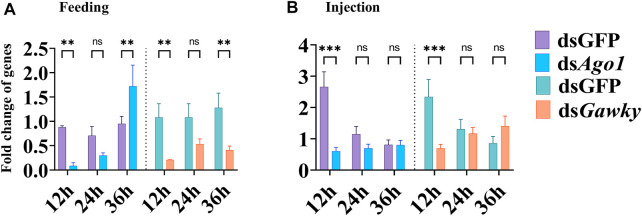
Changes in the expression of *Ago1* and *Gawky* after dsRNA treatment by feeding or injection. The values of the relative expression level of *Ago1* and *Gawky* from the treatment were calculated with those from the control group fed and injected with dsGFP. **(A)** Expression pattern of *Ago1* and *Gawky* after feeding of their dsRNAs. **(B)** Expression pattern of *Ago1* and *Gawky* after injection of their dsRNAs. The bars represent 2^–ΔΔCT^ values (± SD) normalized to the geometrical mean of the expression of two housekeeping genes. The asterisks above the bars designate significant differences (*p < 0.05; **p < 0.01; ***p ≤ 0.001, ns: not significant).

RNAi efficiency was determined after 12, 24, and 36 h of injection and feeding of gene-specific dsRNA and dsGFP. The embryonic injection and oral larval feeding of *Ago1* and *Gawky* dsRNA showed significant transcript knockdown of both genes at 12 h post-delivery of dsRNA, compared to their respective injected and fed dsGFP controls (Figures 3A,B). Reduction in the relative expression of *Ago1* and *Gawky* genes at 24 h post-injection and feeding were non-significant. However, at the longer time period of 36 h post-feeding, the expression level of *Ago1* was recovered and increased, while significant suppression of the target transcript was observed by providing ds*Gawky* compared to the controls with dsGFP (Figure 3A). Furthermore, to measure the possibility of off-target effects, the expression level of non-target genes was estimated. No significant effect on the expression of other Ago family members (*Ago2*, *Ago3*, *Piwi*, and *Aubergine*) was observed after *Ago1* and *Gawky* knockdown (data not shown), which is suggestive of the specificity of *Ago1* and *Gawky* knockdown.

### Knockdown of *Ago1* and *Gawky* Caused Developmental Defects and Severe Mortality in *Z. cucurbitae*

**FIGURE 4:**
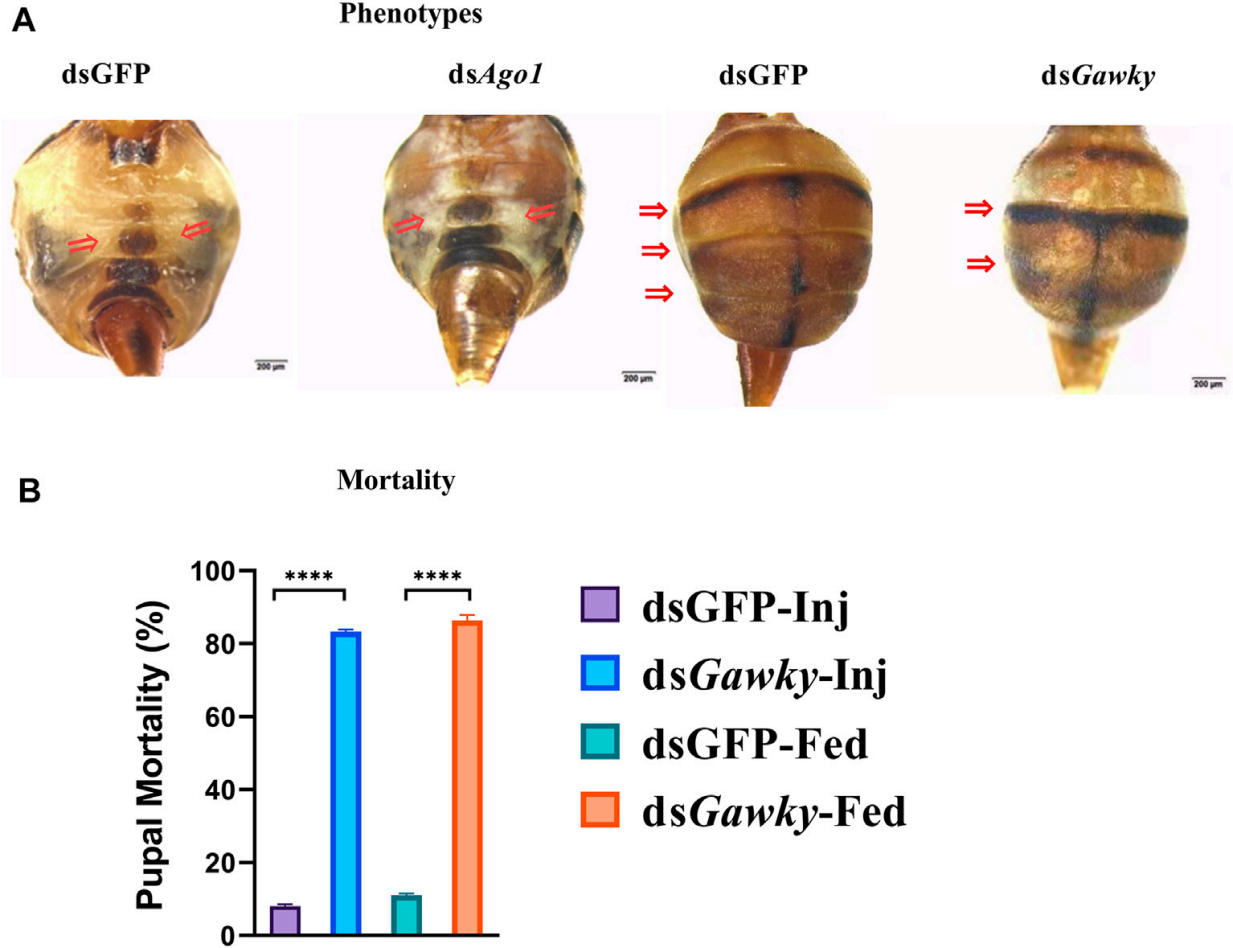
Structural abnormalities and developmental defects after silencing *Ago1* and *Gawky* of *Z. cucurbitae*. 3^rd^ instar larvae were fed dsRNA in the artificial diet and eggs 30 m after egg-laying were microinjected with dsRNA. dsGFP was used as a control. **(A)** Phenotypes of flies delivered with dsRNA of *Ago1*, *Gawky*, and GFP. Arrows represent the loss of pigmentation for ds*Ago1* and the loss of body segmentation for ds*Gawky*. **(B)** Mortality (%) of *Z. cucurbitae* due to silencing ds*Gawky*. The bars represent the mean ± SD with three biological replicates. The asterisks above the bars represent significant differences between the control and treatment groups (****p ≤ 0.001). dsGFP-Inj and ds*Gawky*-Inj represent silencing GFP and *Gawky* by microinjecting their dsRNAs, respectively. dsGFP-Fed and ds*Gawky*-fed represent silencing GFP and *Gawky* by feeding their dsRNAs, respectively.

Silencing *Ago1* and *Gawky* in *Z. cucurbitae via* feeding larvae and microinjecting embryos caused developmental defects. In contrast to the dsGFP control group that had normal yellow ventral segments, adult flies with *Ago1* silencing showed color transition from yellow to white or white ventral patches (Figure 4A). The treatments of ds*Ago1* injection and feeding resulted in adult flies with 38.33 and 46.6% white ventral patches, respectively (Supplementary Tables S2, S3). Severe developmental defects were also observed in the flies with *Gawky* silencing, such as a reduction in abdominal segmentation in relation to the dsGFP-treated control (Figure 4A). About 33.33 and 35.29% abnormal phenotypes were observed with ds*Gawky* injection and feeding, respectively (Supplementary Tables S2, S3). Additionally, compared to dsGFP, *Gawky* silencing presented the highest mortality rate of about 80–85% in both dsRNA delivery groups (Figure 4B). The most efficient mortality occurred in the pupal stage after ingestion and injection of ds *Gawky*. Taken together, these results indicate that *Gawky* is an essential gene for both growth and development of *Z. cucurbitae*, and ds*Ago1* does not affect its viability.

### Effects of *Ago1* and *Gawky* Silencing on Reproduction of *Z. cucurbitae*

**FIGURE 5:**
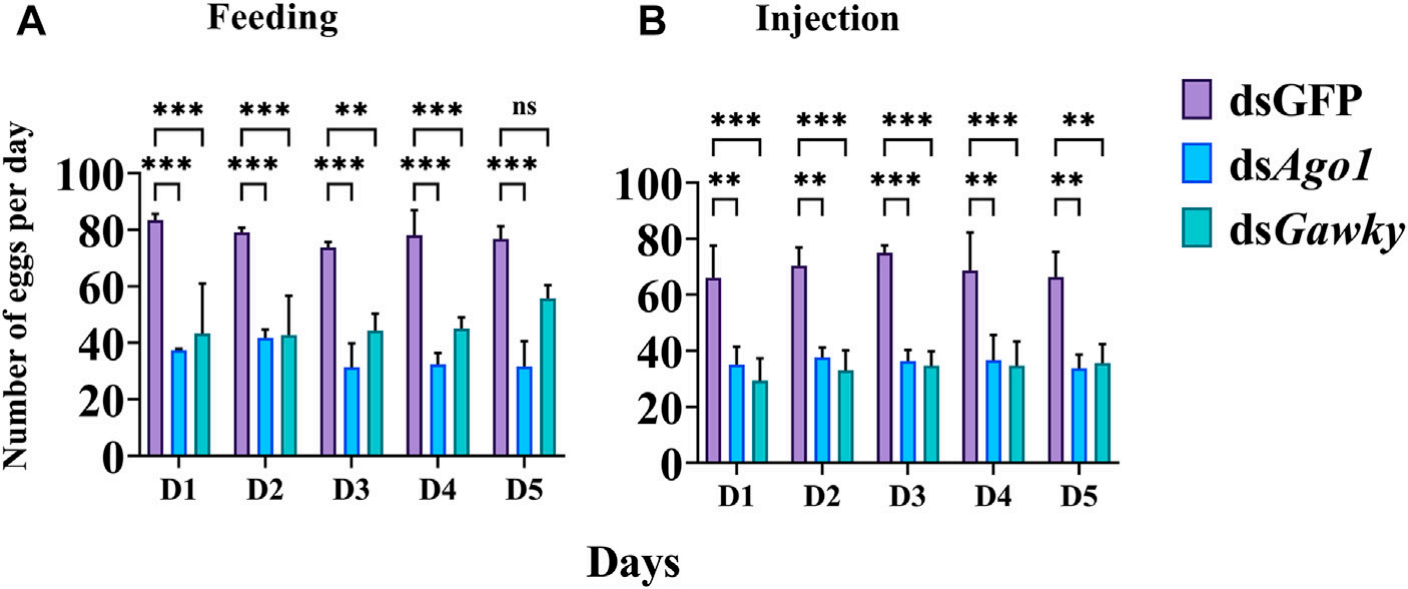
Effects of *Ago1* and *Gawky* RNAi on egg-laying of *Z. cucurbitae*. **(A)** 3^rd^ instar larvae were fed with dsGFP, ds*Ago1*, and ds*Gawky*, respectively. For each treatment, one treated female was crossed with two WT males and the number of laid eggs was recorded for five consecutive days and averaged. **(B)** Embryos were injected with dsGFP, ds*Ago1*, and ds*Gawky*. For each treatment, one treated female was crossed with two WT males and the number of laid eggs was recorded for five consecutive days and averaged. The bars represent the mean ± SD with three biological replicates. The asterisks above the bars represent significant differences between the control and treatment groups (*p < 0.05; **p < 0.01; ***p ≤ 0.001, ns: not significant).

The role of *Ago1* and *Gawky* in reproduction of *Z. cucurbitae* was investigated by genetic crosses. The females and males (9 days old) from both treated groups (feeding and microinjection) were individually crossed with two virgin males and virgin females, respectively. In the control group, one WT male was crossed to two WT females, and one WT female was crossed to two WT males. For the treated groups, one treated male was crossed to two WT females, and one treated female was crossed to two WT males in courtship cages for five consecutive days. Similar crosses were performed for the dsGFP group. The eggs laid by each group were counted daily. No effect on egg-laying was observed when the treated males of ds*Ago1* and ds*Gawky* were crossed with WT females compared to the control group in both dsRNA delivery groups. Egg-laying was significantly reduced (about 60%) in both dsRNA delivery methods when the ds*Ago1*- and ds*Gawky*-treated female flies were crossed with WT males for five consecutive days (Figures 5A,B).

**FIGURE 6:**
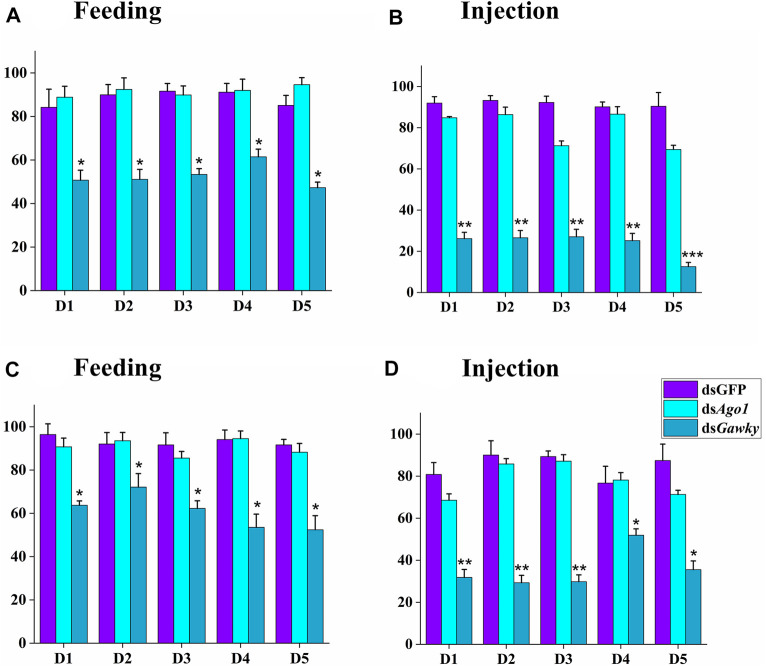
Effects of *Ago1* and *Gawky* RNAi on egg hatching of *Z. cucurbitae*. The larvae hatched from laid eggs in Figure 5 were recorded and the percentages of average numbers of hatched eggs were calculated. **(A)** and **(C)** Feeding treatment. **(B)** and **(D)** Injection treatment. **(A)** and **(B)** In the control group, one dsGFP male was crossed with two WT females, while in the treatment group, one treated male was crossed with two WT females. **(C)** and **(D)** In the control group, one dsGFP female was crossed with two WT males, while in the treatment group, one treated female was crossed with two WT males. Statistical significance was calculated using a one-way ANOVA followed by Tukey’s *post hoc* test. The bars represent the mean ± SD with three biological replicates. The asterisks above the bars represent significant differences between the control and treatment groups (*p < 0.05; **p < 0.01; ***p ≤ 0.001).

To investigate the gene silencing effects of *Ago1* and *Gawky* on reproductive capacity of *Z. cucurbitae*, we further carried out hatching assays of laid eggs. There were no significant differences in egg hatching in both treated methods (feeding and injection) when the treated males of ds*Ago1* were crossed with WT females (Figures 6A,B) or the treated females of ds*Ago1* were crossed with WT males (Figures 6C,D). In both the dsRNA delivery methods, egg hatching was significantly reduced when the ds*Gawky-*treated males and ds*Gawky*-treated females were crossed with WT females and males, respectively. For injection of ds*Gawky*, 60–70% egg hatching reduction was observed in both the treated male and female groups compared to the control group (Figure 6). In view of these results, the reduction in egg hatching was probably due to the developmental defects observed in the physiology of the ds*Gawky* treated flies, where there was no effect of ds*Gawky* on reproductive tissues of *Z. cucurbitae*.

### *Ago1* Silencing Caused Ovarian Defects

**FIGURE 7:**
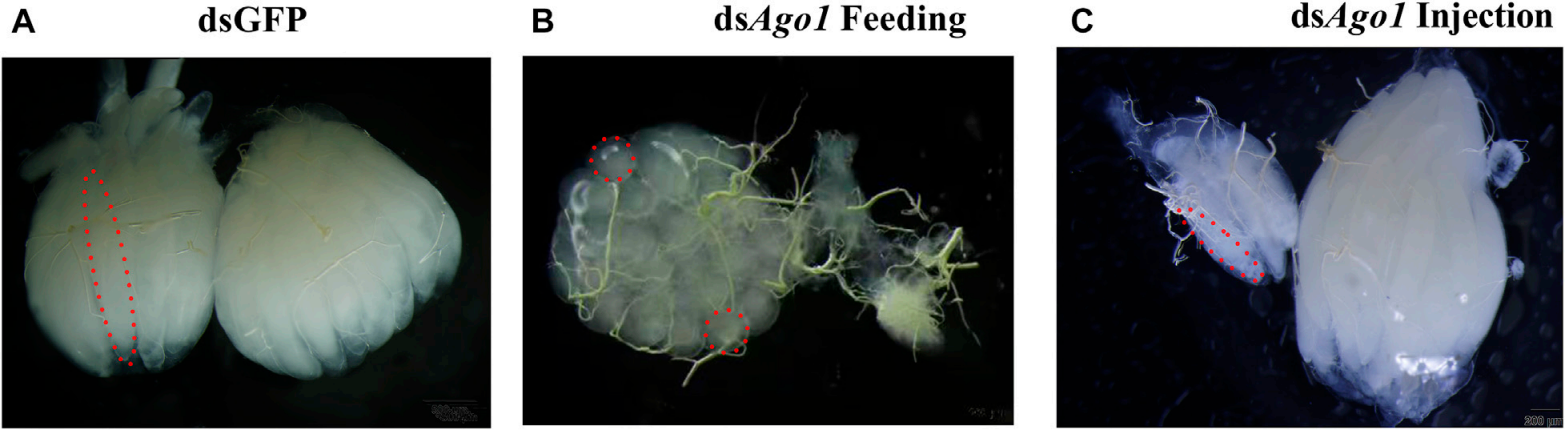
Ovarian defects in the 14-day-old ovary after *Ago1* silencing. Ovaries were dissected separately in 1 × PBS (pH 7.4) and visualized under a binocular stereomicroscope. Ovarioles are indicated by dashed red lines. **(A)** Normal ovaries in dsGFP control. **(B)** ds*Ago1* feeding. One spheroid of the ovary had tiny round ovarioles, while the development of the other one was significantly arrested. **(C)** ds*Ago1* injection. One spheroid of the ovary had long but few ovarioles compared to the other one which developed normally.

After the effects on reproductive capacity with *Ago1* and *Gawky* silencing were observed, we speculated that these genes are probably required for reproductive tissue development. To test this assumption, reproductive tissues of 14-day-old females and males after genetic crosses were dissected to check the effects of target genes on ovary and testis morphology. *Ago1* silencing caused ovarian abnormalities, such as undifferentiated and undeveloped ovaries, decreased number of ovarioles, and abnormal oviposition in female adults (Figure 7). Different phenotypes in ovaries were observed after feeding and injection of ds*Ago1*. Feeding of ds*Ago1* affected the ovary morphology. One ovary from each pair has an undifferentiated and tiny round ovariole, while the other has no reproductive tissue, and its development was completely arrested (Figure 7B). Egg count was also significantly reduced with ds*Ago1* compared to dsGFP. After injection of ds*Ago1*, one spheroid of the ovary was fully developed with long ovarioles, while the other one had few long ovarioles compared to the normal ovary in the control group (Figure 7C). No effect was observed on the morphology of testes of *Z. cucurbitae* after the *Ago1* knockdown. These results suggest that *Ago1* plays a vital role in the reproduction of *Z. cucurbitae* females. No testis and ovary malformation were observed, and each ovary contained normal ovarioles in the ds*Gawky* treated flies, similar to the dsGFP control group. Our findings are partially coherent with our speculation that decrease in egg number is due to ovarian defects in the ds*Ago1* treated females. However, reduced number of egg hatchability are not associated with these reproductive tissues in the ds*Gawky*-treated flies.

## Discussion

The management of *Z. cucurbitae* is becoming increasingly difficult due to the rapid development of pesticide resistance. This study aims to investigate the roles of *Ago1* and *Gawky* in *Z. cucurbitae*, which might be used as potential targets for developing innovative pest management strategies against this insect pest. We have amplified the open reading frame sequences of *Ago1* and *Gawky* in *Z. cucurbitae*. Phylogenetic analysis revealed that these proteins are conserved in different fruit fly species, including *D. melanogaster*. Our findings show that *Ago1* has two functional domains, PAZ and PIWI, which are very similar to its homologs in other insects, such as *Z. cucurbitae*, *D. melanogaster*, and *T. castaneum* (Xu et al., 2013; Yang et al., 2021b). *Gawky* contains two functional domains, RRM and the M domain. These domains are the active centers that execute the cutting of target mRNA (Cieplak-Rotowska et al. 2017: BoulétreauMerle et al.,1982; Inada et al., 2015).

Temporal and spatial expressions of *Ago1* and *Gawky* were determined in different developmental stages and tissues of *Z. cucurbitae*. The high expression of *Ago1* and *Gawky* indicates that these genes have key roles in development and sexual maturation of *Z. cucurbitae* females. According to temporal analysis, *Ago1* was expressed in all developmental stages with the highest amount in eggs followed by an adult. The expression level of *Ago1* was the highest in the ovary followed by the fat body in the spatial expression. *Ago1* in *Z. cucurbitae* shows a consistent mRNA expression pattern with its homologs (Gao et al., 2020). *Gawky* expression was the highest in eggs followed by an adult in temporal expression, while in spatial expression, its level was the highest in the head of *Z. cucurbitae*. These results indicate that *Gawky* may play a key role in the nervous system, possibly due to the diverse activities of their ligands. The high expression of *Ago1* in the ovary and *Gawky* in the head implies that these genes play important roles in these tissues. Previous studies have shown that *Ago1* is highly expressed in *Drosophila* oocytes, while in *B. mori*, it is mainly expressed in the ovary, testis, midgut, and head (Wu et al., 2021; Yang et al., 2021; Zhao et al., 2021). These various expression patterns may be due to the specific functions of *Ago1* genes in different insect species.

In this study, different responses of both genes were detected after their silencing *via* feeding and injection. The expression level of *Ago1* decreased between 12 and 24 h, while it was recovered at 36 h of post-feeding, which demonstrated the activation of RNAi response. Significant reduction of *Ago1* at an early stage indicates its effectiveness at this stage to cause loss of pigmentation and ovarian defects in *Z. cucurbitae*. A similar response was observed to *Ago2* and *Dicer2* upon dsRNA exposure in *Anastrepha fraterculus* and ds*IDGF4_1* feeding in *Z. cucurbitae* (Dias et al., 2019; Ahmad et al., 2021). In addition to the core RNAi machinery genes, such phenomena have been extensively noticed in several other mechanisms of target gene silencing including dsRNA uptake and degradation (Zhu and Palli 2020). This difference in response to ds*Ago1* may provide an excellent tool to further define its resistance to insect pests.

The function of *Ago1* in the reproduction and development of *Z. cucurbitae* was explored with dsRNA oral feeding in 3^rd^ instar larvae and injection into embryos. Compared to the dsGFP group, significant transcript reduction was observed after 12 h, and there was no significant decrease in *Ago1* and *Gawky* transcription 24 h after injection and feeding of their dsRNA. *Ago1* dsRNA in both delivery methods leads to ovarian abnormalities. The ovarian morphology was defective with the malformed and reduced number of ovarioles in ds*Ago1*-fed flies. About 50% of *Z. cucurbitae* females after dsRNA treatment had unilateral ovaries. The shape of the ovarioles was rounded, not elongated, which might be due to insufficient supply of nutrients or decrease in vitellogenin in larval fat body. The fat body plays an essential role in female reproduction and responds to the juvenile hormone by synthesizing yolk protein, and supporting oocyte maturation and ovarian development (Wyatt and Davey 1996; Wu et al., 2020). Similar phenotypes were observed in RNAi-mediated knockdown of *anne boleyn*, *bin3*, *blot*, *kirre*, *slim*, *VACht*, and *zfh1* in *D. melanogaster*, where 66% of flies had one ovary or no visible reproductive tissues (Lobell et al., 2017). It will be interesting to examine whether the underlying mechanism that controls these genes also impacts *Ago1*. *Ago1* depletion at the embryonic stage resulted in 38.33% comparable phenotypes in ovarian size and ovariole number with dsGFP. The ovarioles were lengthy and well-differentiated, similar to the control. The number of laid eggs from the ds*Ago1* microinjection females was comparatively higher than the ds*Ago1* feeding females but significantly lower than the dsGFP control females. This could explain the difference in the dsRNA delivery stage and the effects of continuous dsRNA exposure to larvae *via* feeding, which improves silencing efficiency. These findings strongly support the hypothesis that ovariole number is a quantitative attribute that influences the number of eggs laid by a female fly (Boulétreau-Merle et al., 1982; Lobell et al., 2017).

*Ago1* knockdown in embryos and larvae identifies its impact on female reproductive morphology and fitness, confirming its involvement in ovariole development. We report for the first time that *Ago1* silencing at early developmental stages affects ovariole development in *Z. cucurbitae*. When *B. dorsalis* adult females were injected with ds*Ago1*, the ovarian growth was stopped and both ovaries were substantially smaller but had no effect on ovariole number, shape, or oviposition (Yang et al., 2021). *Ago1* knockdown in *L. migratoria* caused decrease of the *vgA* transcript in the fat body, which arrests ovary development and oocyte maturation, and results in short terminal oocytes (Song et al., 2013). These results suggest that the effects of *Ago1* silencing on egg production and ovarian morphology fluctuates with the RNA interference method, dsRNA stability, delivery stage, or different insect species. The ds*Ago1* treatments at both stages do not affect the viability *of Z. cucurbitae*, which is similar to *Ago1*-depleted *L. migratoria* (Song et al., 2013). However, knocking down of *Ago1* in *Diabrotica virgifera* at the larval stage delayed its development and induced mortality (Camargo et al., 2018). In insects, pigmentation fluctuates even within the same species (Massey and Wittkopp 2016). miRNA-8 is involved in pigmentation in *D. melanogaster* (Kennell et al., 2012) and *Ago1* mutation disrupts cuticle pigmentation of *Ostrinia furnacalis* (You et al., 2019). In the present study, we have observed that knockdown of *Ago1* also causes discoloration in lower ventral abdominal segments. This may be due to the downregulation of microRNA and pigmentation-related genes. Our study is consistent with these results and reports the involvement of *Ago1* in the pigmentation of *Z. cucurbitae*, which is suggestive of a conserved role of *Ago1* in the pigmentation of insects. Thus, it will be of considerable interest to uncover the underlying mechanism behind it in the future.

*Gawky* is a crucial component of the cytoplasmic processing body and the miRNA repressor complex. It aids P-body formation and serves as a scaffold for mi-RISC and other RNA decay factors (Braun et al., 2013; Niaz and Hussain 2018). *Gawky* is primarily involved in cytoplasmic post-transcriptional regulation, such as deadenylation, decapping, or degradation of RNAs, as an RNA decay factor (Piao et al., 2010; Jia et al., 2019; Jia et al., 2021). In the present work, we have investigated the effects of injection and oral feeding of *Gawky* dsRNA in *Z. cucurbitae* embryos and 3^rd^ instar larvae. *Gawky* silencing resulted in the highest mortality at the pupal stage, loss of body segmentation, malformed wings, reduction in mobility, and low egg count in female adults. The changes in *Gawky* gene expression and mortality rate suggest its role in the developmental process of *Z. cucurbitae*. The lethal effect of *Gawky* knockdown might be associated with its central role in the miRNA regulation required for insect development and survival. miRNA is required for a wide range of functions such as cellular proliferation, oogenesis, metabolic homeostasis, embryonic development, and cell death (Brennecke et al., 2003). Similar to our findings, *Gawky* disruption induces growth arrest and embryonic mortality in mice (Guo et al., 2017). In *Euschistus heros*, seven and 14 days post-injection of ds*Gawky* in nymph caused 50–90% mortality, respectively (Castellanos et al., 2019), which is consistent with our result that knockdown of the *Gawky* gene causes severe mortality. Furthermore, low egg count and egg hatchability were significantly reduced when *Gawky* in *Z. cucurbitae* was silenced. Similarly, depletion of the *Gawky* gene in early *Drosophila* embryos with the maternal-Gal4–shRNA system caused 100% abnormal oogenesis, fused filaments, and low egg count (Staller et al., 2013).

In conclusion, we have comprehensively measured the expression pattern of *Ago1* and *Gawky* by qRT-PCR, and analyzed their functions in *Z. cucurbitae* using the RNAi method. This study shows that *Ago1* and *Gawky* are involved in ovarian development, reproduction, and viability in *Z. cucurbitae*. Further investigation of their functions is expected to provide insight into the roles of miRNA in reproduction and development, and help understand the regulatory mechanism of these two genes. Owing to their comprehensive involvement in the development of insects, they can be chosen as ideal targets for genetic engineering pest control, such as improving the sterile insect technique (SIT).

## Data Availability

The original contributions presented in the study are included in the article/Supplementary Material; further inquiries can be directed to the corresponding authors.
