# Supplementary material for: Argonaute1 and Gawky Are Required for the Development and Reproduction of Melon fly, Zeugodacus cucurbitae
Source: Front Genet. 2022 Jun 23;13:880000. doi: 10.3389/fgene.2022.880000 (PMC9260231; doi:10.3389/fgene.2022.880000)
Supplement: Supplementary file 1 [file DataSheet1.docx]

Supplementary Materials

| Table S1. Argonaute protein sequences used for Phylogenetic analysis. | | | |
| --- | --- | --- | --- |
| No. | Species | Name used in P. tree | Accession Number |
| 1 | *Zeugodacus cucurbitae* | ZcAgo1 | XP_011189086.1 |
| 2 | *Bactrocera dorsalis* | BdAgo1 | XP_011201158.1 |
| 3 | *Bactrocera oleae* | BoAgo1 | XP_014085215.1 |
| 4 | *Drosophila obscura* | DoAgo1 | ALC79965.1 |
| 5 | *Drosophila immigrans* | DiAgo1 | ALC79964.1 |
| 6 | *Ceratitis capitata* | CcAgo1 | XP_004529840.1 |
| 7 | *Drosophila melanogaster* | DmAgo1 | ABD61617.1 |
| 8 | *Drosophila subobscura* | DsAgo1 | ALC79966.1 |
| 9 | *Drosophila simulans* | DsiAgo1 | EDX06986.1 |
| 10 | *Drosophila subsilvestris* | DsvAgo1 | ALC79967.1 |
| 11 | ***Locusta migratoria*** | **LmAgo1** | **KF006338** |
| 12 | *Zeugodacus cucurbitae* | ZcGawky | XP_011195144.1 |
| 13 | *Bactrocera oleae* | BoGawky | XP_014093589.1 |
| 14 | *Bactrocera latifrons* | BlGawky | XP_018799543.1 |
| 15 | *Bactrocera tryoni* | BtGawky | XP_039968083.1 |
| 16 | *Drosophila virilis* | DvGawky | XP_032290165.1 |
| 17 | *Drosophila novamexicana* | DnGawky | XP_030566498.1 |
| 18 | *Drosophila mojavensis* | DmoGawky | XP_032588792.2 |
| 19 | *Drosophila melanogaster* | DmGawky | NP_726596.1 |
| 20 | *Drosophila albomicans* | DaGawky | XP_034112019.1 |
| 21 | *Drosophila suzukii* | DsGawky | XP_016944114.1 |
| 22 | *Bactrocera dorsalis* | BdGawky | XP_029406091.1 |
| 23 | *Ceratitis capitata* | CcGawky | XP_004533549.3 |

**Table S2**: Abnormal flies observed post-injection of gene-specific dsRNA.

| Treatment | No. of injected eggs | No. of hatched larvae | Hatchability (%) | No. of adults emerged (%) | No. of flies observed | No. of abnormal flies | Percentage of abnormal flies (%) |
| --- | --- | --- | --- | --- | --- | --- | --- |
| dsGFP | 900 | 505 | 61.2 | 91.08 | 60 | 0 | 0 |
| ds*Ago1* | 900 | 497 | 60.2 | 86 | 60 | 23 | 38.33 |
| ds*Gawky* | 900 | 650 | 78.7 | 9.23 | 60 | 20 | 33.33 |

**Table S3**: Abnormal flies observed post-feeding of gene-specific dsRNA.

| Treatment | No. of fed larvae | No. of pupae emerged | No. of adults emerged (%) | No. of flies observed | No. of abnormal flies | Percentage of abnormal flies (%) |
| --- | --- | --- | --- | --- | --- | --- |
| dsGFP | 180 | 158 | 93.03 | 100 | 0 | 0 |
| ds*Ago1* | 180 | 131 | 87.78 | 60 | 28 | 46.6 |
| ds*Gawky* | 180 | 125 | 13.6 | 17 | 6 | 35.29 |

**Table S4**: Primers used for cloning, dsRNA and qRT-PCR amplification.

| **Gene** | **Primer** | **Sequence** | **Size** |
| --- | --- | --- | --- |
| ***Ago1*** | whole seq-F  whole seq-R  dsRNA –F  dsRNA –R  qpcr-F  qpcr-R | CCAAGCAGCGTTCGTACAAC  CTTATAAGAATTGCTTAATCGCACG  GGATCCTAATACGACTCACTATAGGCCAAGCAGCGTTCGTACAAC  GGATCCTAATACGACTCACTATAGGGATTCGCTTCGTCCACATCAC  CTTTCGTTGGCGTTGACC  GGCTTCGTCTGGATTACG | 1388  407  138 |
| ***Gawky*** | whole seq-F  whole seq-R  dsRNA –F  dsRNA –R  qpcr-F  qpcr-R | CGTCTACTAAAAGCGCTATC  CTCGAACATTACCTTTAGCTTCCAC  GGATCCTAATACGACTCACTATAGGCGTCTACTAAAAGCGCTATC  GGATCCTAATACGACTCACTATAGGCCCTCCAACAGTTCGAGATAC  GCCTTCGATTTCCTCACAC  CACGTTGCAACAACCAATTC | 2836  440  136 |
| ***EFα1-rt*** | qpcr-F  qpcr-R | CGTTGGTGTCAACAAGATGG  TGCCTTCAGCATTACCTTCC | 230 |
| ***Actin*** | qpcr-F  qpcr-R | GACTCGTACGTCGGTGAC  CTGTGTCATCTTCTCACGG | 200 |
| ***GFP*** | dsRNA-F  dsRNA-R  RT-PCR-F  RT-PCR-R | TAATACGACTCACTATAGGGCAGTGGAGAGGGTGAA  TAATACGACTCACTATAGGGTTGACGAGGGTGTCTC  CAGTGGAGAGGGTGAAG  TTGACGAGGGTGTCTC | 711 |

#
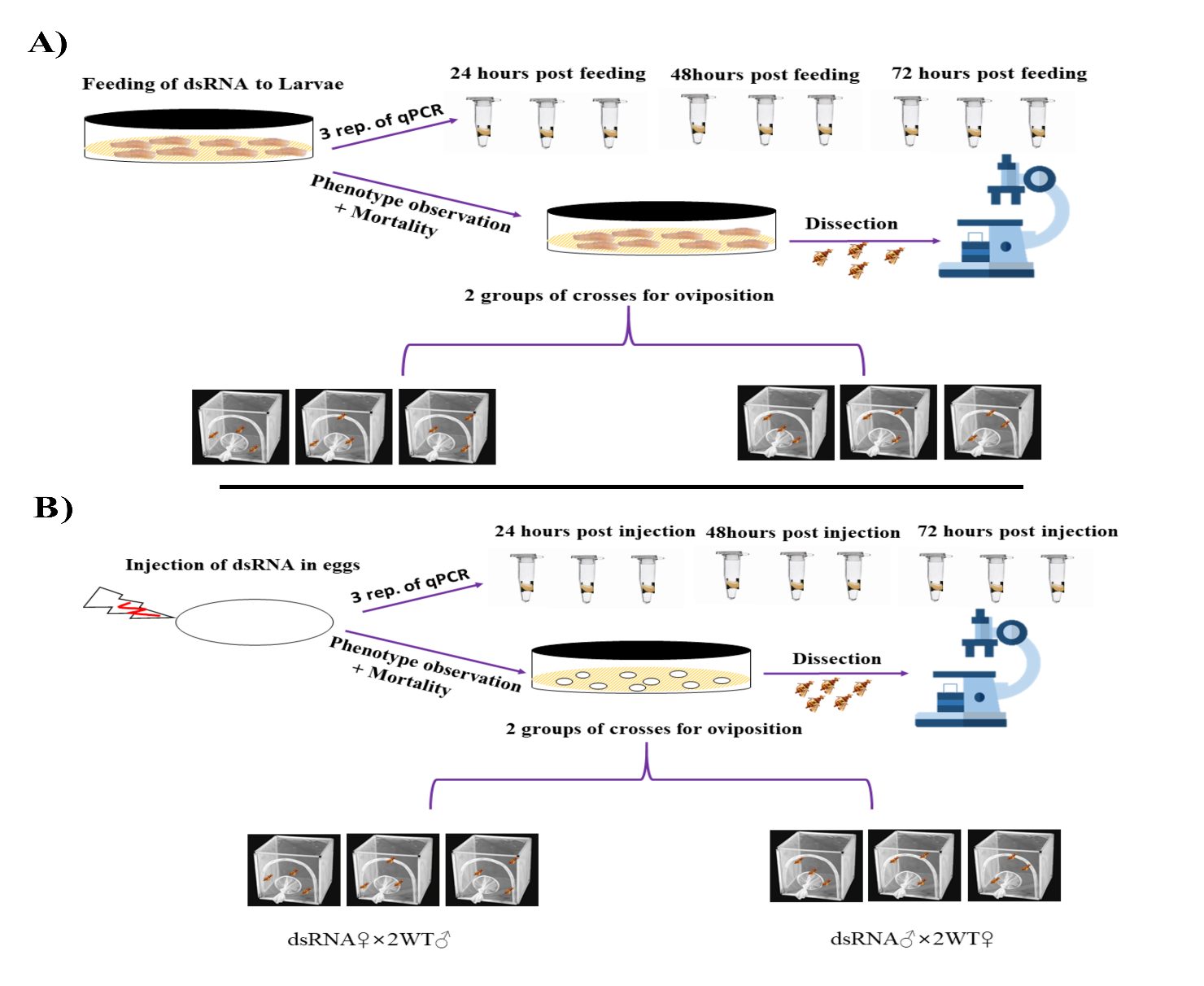


**Figure S1**: Experimental set-up for feeding (A) and microinjection (B) of *Ago1* and *Gawky*. Microinjection experiment *n*=900 (three replicates of 300 eggs each). Feeding experiment: *n*=180 (three replicates of which 60 individuals in each replicate). In feeding experiment, 3^rd^ instar larvae were fed with dsRNA of each gene mixed in artificial diet.


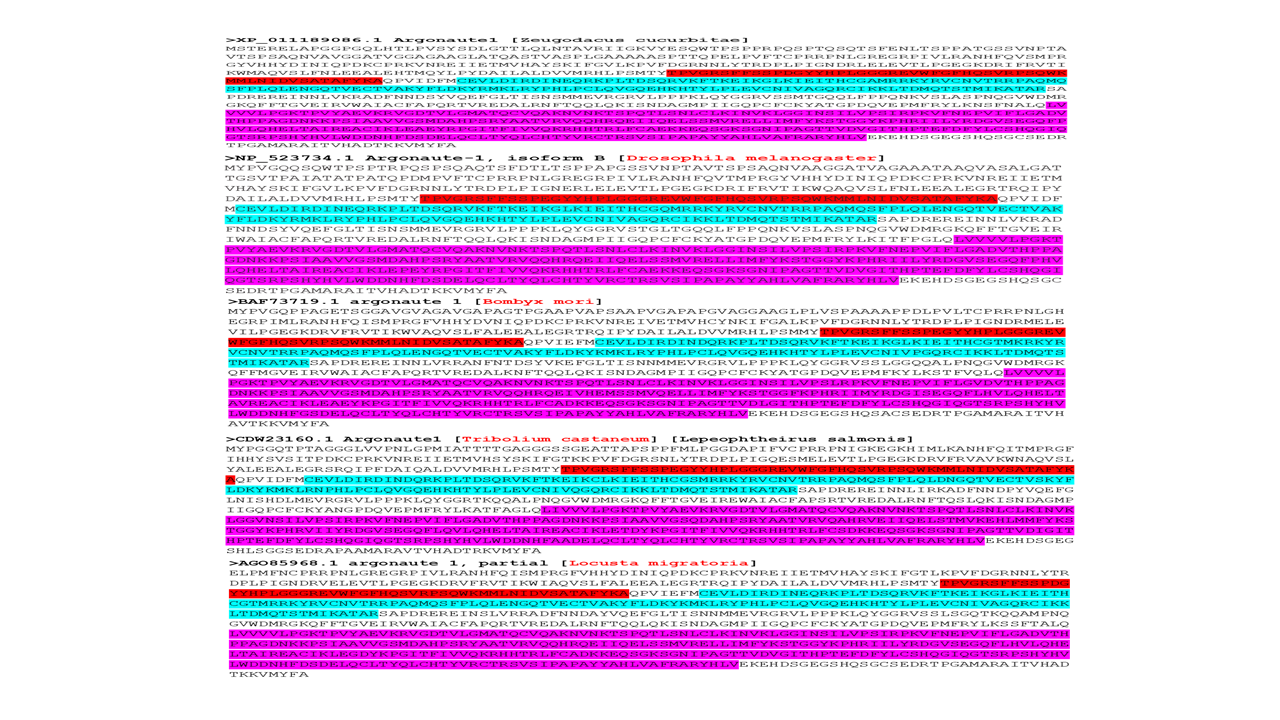


**Figure S2:** Comparison of the Argonaute1 domains in *Z. cucurbitae* with *D. melanogaster*, *B. mori*, *T. castaneum* and *L. migratoria*. DUF, PAZ and PIWI domains are highlighted with red, aqua and purple colors, respectively.


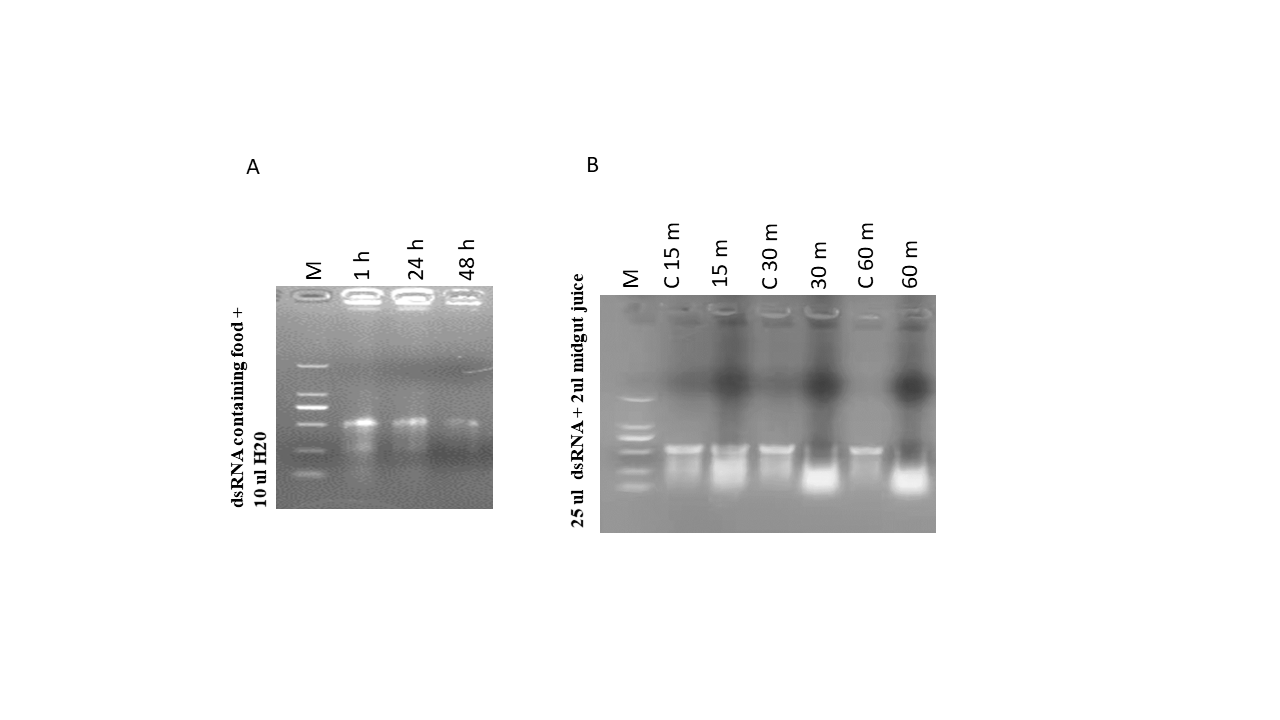


**Figure S3:** Ex vivo dsRNA degradation assays from midgut juice and artificial diet. dsRNA was diluted in midgut juice and artificial food. Stability of dsRNA was examined in (A) artificial diet and (B) midgut juice compared with the control (dsRNA-containing ddH_2_O instead of midgut juice) at different time intervals. C represents control at different time periods.
